# The Impact of Vitamin D on Androgens and Anabolic Steroids among Adult Males: A Meta-Analytic Review

**DOI:** 10.3390/diseases12100228

**Published:** 2024-09-25

**Authors:** Ahmed Abu-Zaid, Saleh A. K. Saleh, Heba M. Adly, Saeed Baradwan, Abdullah M. Alharran, Mshal Alhatm, Mooza M. Alzayed, Muteb N. Alotaibi, Abdulbadih Rabih Saad, Hessa Mohammed Alfayadh, Mohammed Abuzaid, Osama Alomar

**Affiliations:** 1 Department of Biochemistry and Molecular Medicine, College of Medicine, Alfaisal University, Riyadh 11533, Saudi Arabia; 2 Department of Biochemistry, Faculty of Medicine, Umm Al-Qura University, Makkah 24382, Saudi Arabia; 3 Department of Community Medicine and Pilgrims Healthcare, Faculty of Medicine, Umm Al-Qura University, Makkah 24382, Saudi Arabia; 4 Department of Obstetrics and Gynecology, King Faisal Specialist Hospital and Research Center, Jeddah 23433, Saudi Arabia; 5 College of Medicine and Medical Sciences, Arabian Gulf University, Manama 329, Bahrain; 6 College of Medicine, Alfaisal University, Riyadh 11533, Saudi Arabia; 7 College of Medicine, Almaarefa University, Riyadh 13713, Saudi Arabia; 8 Department of Obstetrics and Gynecology, Al Birk General Hospital, Al Birk 63525, Saudi Arabia; 9 Department of Obstetrics and Gynecology, King Faisal Specialist Hospital and Research Center, Riyadh 11564, Saudi Arabia

**Keywords:** vitamin D, testosterone, free androgen index, reproductive hormones, estradiol

## Abstract

Background: Recent studies indicate that vitamin D impacts male reproductive function, with deficiency linked to infertility. This review evaluates the effect of vitamin D supplementation on male fertility, focusing on total testosterone, free testosterone, the free androgen index (FAI), follicle-stimulating hormone (FSH), luteinizing hormone (LH), sex-hormone-binding globulin (SHBG), and estradiol. Methods: We systematically searched Medline, Web of Science, Cochrane Library, and Scopus from their inception until July 2024 for randomized controlled trials (RCTs) involving adult males. The primary focus of these studies was on reproductive hormone parameters, analyzed using a random-effects meta-analysis and weighted mean difference (WMD). Evidence quality was assessed using ROB2 and GRADE. Meta-regression and dose–response analyses were performed. Results: Seventeen studies met the criteria for quantitative analysis. Vitamin D supplementation significantly increased total testosterone levels (WMD 0.38, 95% CI 0.06–0.70, n = 15, I^2^ = 67.03). However, it had no significant effect on other hormone parameters: free testosterone (WMD 0.00, 95% CI −0.02–0.03, n = 9, I^2^ = 48.12), FSH (WMD −0.02, 95% CI −0.57–0.53, n = 7, I^2^ = 48.72), LH (WMD −0.09, 95% CI −0.30–0.12, n = 8, I^2^ = 0.00), SHBG (WMD 0.73, 95% CI −1.14–2.61, n = 10, I^2^ = 69.05), FAI (WMD −0.92, 95% CI −2.12–0.27, n = 6, I^2^ = 0.00), and estradiol (WMD −0.02, 95% CI −2.95–2.92, n = 5, I^2^ = 20.63). Conclusion: This meta-analysis shows that vitamin D supplementation may increase total testosterone levels in men. However, further well-designed RCTs are needed to determine vitamin D’s effects on other reproductive hormone parameters.

## 1. Introduction

It is acknowledged that vitamin D is crucial for the absorption of both phosphate and calcium, which are essential for preserving a healthy skeletal system [1]. The primary source of this secosteroid is the skin, where ultraviolet (UV) radiation converts the cholesterol precursor, 7-dehydrocholesterol, into vitamin D3 (cholecalciferol) [2]. The serum level of 25-hydroxyvitamin D [25(OH)D], a stable metabolite of vitamin D, is considered to be a trustworthy biomarker of vitamin D levels [3,4]. Recent discoveries have provided novel understandings of the biological functions of vitamin D and its capacity to lower the hazard of various chronic disorders [5], including cardiovascular disease [6], infectious and autoimmune diseases [7], and cancer [7]. Therefore, vitamin D may have broader physiological implications, influencing multiple organ systems and metabolic pathways [8,9].

The expression of vitamin D receptors (VDRs) and vitamin D-metabolizing enzymes (VDMEs) in the testes, male reproductive system, and human sperm indicates that vitamin D is likely important for male reproductive functions, including spermatogenesis and the maturation of human sperm [10].

Male infertility is an increasing concern in developed nations [11], accounting for 50% of global infertility cases [12]. Genetic disorders, varicocele, genital infections, systemic illnesses, and environmental influences are recognized causes of male infertility. Nonetheless, approximately 30–40% of cases involve unidentified factors [13].

In recent years, dietary antioxidants have garnered significant consideration due to their potential function in enhancing male fertility [14]. One such nutritional factor is vitamin D, and deficiency in this is acknowledged as a sizable public health concern [15]. Substantial positive correlation between 25(OH)D and testosterone was noted in many studies [16,17,18,19]. We remain without an accurate molecular understanding of the relationship between vitamin D and testosterone. However, the level of vitamin D receptors and associated metabolic enzymes (such as CYP2R1) in the testes—particularly in Leydig cells—as well as in the seminal vesicles, epididymis, prostate, and the sperm head area underscores the significant role of vitamin D in male fertility and reproductive health [14]. Hammoud et al. demonstrated that both high serum vitamin D concentrations (≥50 ng/mL) and low serum vitamin D concentrations (<20 ng/mL) can be negatively associated with semen parameters [20]. In a retrospective investigation, vitamin D was positively correlated with testosterone and the free androgen index (FAI) score, while it showed an opposite relationship with sex-hormone-binding globulin (SHBG) [21]. Abbasihormozi et al. failed observe any link between serum vitamin D concentrations, semen quality, and reproductive hormones such as luteinizing hormone (LH), follicle–stimulating hormone (FSH), and free testosterone in normozoospermic men. However, vitamin D levels were positively correlated with sperm motility in men with oligoasthenoteratozoospermia (OAT) [22]. Progressive motility, a key indicator of sperm quality during the analysis of semen, predicts the likelihood of success for naturally occurring conception as well as intrauterine insemination (IUI) [23].

Vitamin D3 has promising effects on these parameters, indicating its potential as a cost-effective tool in infertility treatments and assisted reproductive technologies (ART) [24]. Although numerous reviews have been conducted on the topic, a consensus regarding the contribution of vitamin D to male fertility remains elusive. Additionally, investigations involving human subjects have been conducted in recent years. Therefore, the present study seeks to explore the effect of vitamin D supplementation on male reproductive hormones, specifically targeting androgens and related reproductive hormones in adult men. The investigation will focus on key parameters such as total and free testosterone, the FAI, FSH, LH, SHBG, and estradiol. This focus addresses the inconsistent findings in the existing research.

## 2. Methods

### 2.1. Study Protocol

A systematic review and meta-analysis following the guidelines of the Preferred Reporting Items for Systematic reviews and Meta-Analyses (PRISMA) [25] was conducted and was registered in the International Prospective Register of Systematic Reviews (PROSPERO) database (ID: CRD42024563097).

### 2.2. Search Approach

We conducted a thorough systematic review of the literature to examine the impacts of vitamin D supplementation on androgens and reproductive hormones in men. The search, carried out in July 2024, included multiple databases such as the Cochrane Central Register of Controlled Trials (CENTRAL), Web of Science, PubMed, and Scopus, utilizing terms related to androgens, male reproductive hormones, and various forms of vitamin D. The complete search strategy and database-specific syntaxes are detailed in Supplementary File S1. Furthermore, we reviewed reference lists from original studies, meta-analyses, relevant reviews, and congress abstracts to identify any additional eligible studies. Despite extending the search to Google Scholar, no new relevant articles were found beyond those identified through PubMed.

### 2.3. Study Choice

To be considered in the analysis, studies had to meet the following conditions: they needed to involve male human subjects; focus on androgens and reproductive hormones such as testosterone, SHBG, FSH, LH, dehydroepiandrosterone (DHEA), and estradiol; use vitamin D supplementation as an intervention for at least one week; and provide the average and standard deviation of the variables pre- and post-supplementation. The studies also had to follow a prospective randomized controlled trial (RCT) design. We excluded studies that were case studies, cross-sectional studies, cohort studies, reviews, letters, editorials, or supplementary articles. If multiple publications stemmed from the same study, the one with the longest follow-up time was included. Studies were also excluded if they lacked an appropriate controlled design, involved multivitamin or multimineral supplements including vitamin D, or had overlapping participants with other studies. Non-RCT studies and those without explicit methods were also omitted.

### 2.4. Data Extraction and Quality Evaluation

This study incorporated data extracted from RCTs, including details on the various baseline characteristics of the studies and patients. Two independent reviewers evaluated the studies based on their title, abstract, and full text, resolving disagreements through consensus and consulting a third reviewer if necessary. They extracted participant characteristics, intervention details, and outcome measures using a specially developed data extraction form. Missing or incomplete data were addressed by contacting the study authors via email or referring to previous analyses. This study’s quality evaluation was completed using the Cochrane Risk of Bias assessment tool, version 2 [26].

### 2.5. Statistical Analysis

We evaluated the influence of vitamin D supplementation on the following outcomes and parameters in men: (1) total testosterone; (2) free testosterone; (3) FAI; (4) FSH; (5) LH; (6) SHBG; and (7) estradiol. The meta-analysis was only conducted for variables of interest that were reported in at least three study arms. The dose of vitamin D intake per day was calculated from the vitamin D intake of monthly and weekly interventions. Weighted mean differences (WMDs) and their corresponding SDs were estimated using the DerSimonian and Laird random-effects model. Statistical heterogeneity was examined with the Cochran’s Q test and the I^2^ statistic (I^2^). Subgroup analyses were conducted by health status (infertile/reproductive disorders vs. healthy men), the duration of intervention (≤12 wk, >12 wk), baseline vitamin D levels (not deficient, deficient), the dosage of vitamin D supplementation (≤4000 IU/d, >4000 IU/d), and the age of subjects to determine the potential sources of heterogeneity. We also performed meta-regression to detect the sources of heterogeneity. Sensitivity analyses were performed by excluding one study at a time. Publication bias was assessed by the visual inspection of funnel plots of outcomes, and plot symmetry was assessed statistically using Egger’s and Begg’s methods. Dose–response analysis was conducted for all interested variables, assessing the dose and duration of vitamin D supplementation. All statistical analyses were performed using STATA version 17.0 (STATA Corp. College Station, TX, USA). Two-sided *p* values < 0.05 were considered significant.

## 3. Results

### 3.1. Summary of Study Characteristics

**Figure 1:**
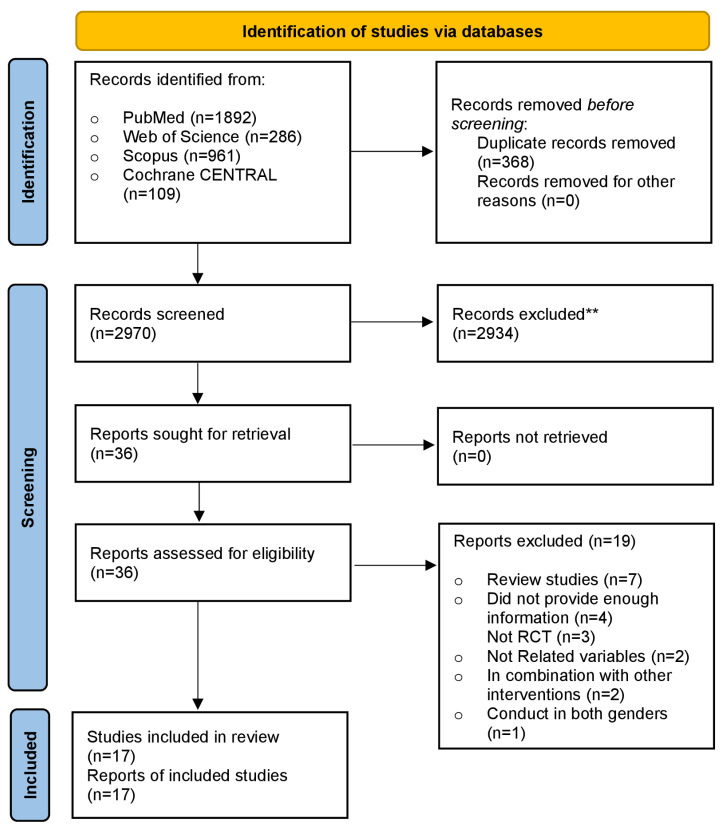
PRISMA flow diagram of included studies. ** Records excluded based on screening of titles and abstracts.

A flow diagram outlining the search of information sources and study selection is presented in Figure 1. Finally, 17 RCTs [24,27,28,29,30,31,32,33,34,35,36,37,38,39,40,41,42] were incorporated into the meta-analysis, accounting for 1774 men (957 in the intervention group and 817 in the control group). The trial’s main features are summed up in Table 1. Sample sizes in the included RCTs fluctuated from 23 to 307 participants, and the mean participant age fluctuated from 20 to 72 years. Seven studies focused on infertile men or those with reproductive disorders, six studies included healthy males, two studies involved patients with severe vitamin D deficiencies, one study examined heart failure patients, and one study focused on overweight men. The dosage of supplementary vitamin D in the treated arms of the 17 included trials varied from 580 to 8500 IU/day. The period of vitamin D supplementation ranged from as low as 8 weeks to as high as 96 weeks.

### 3.2. Pooled Results from Meta-Analysis

**Figure 2:**
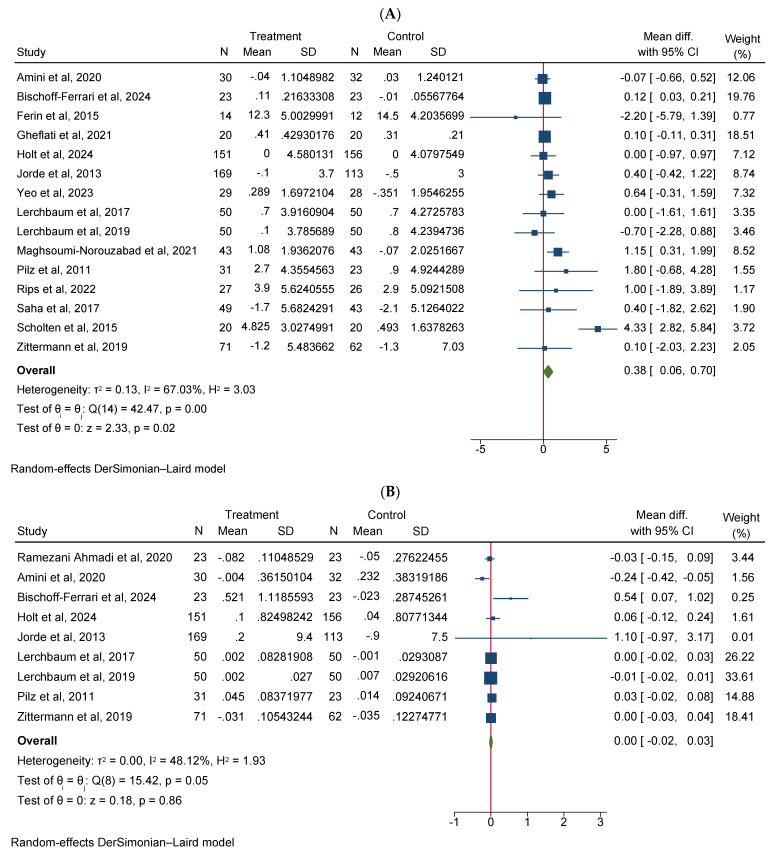

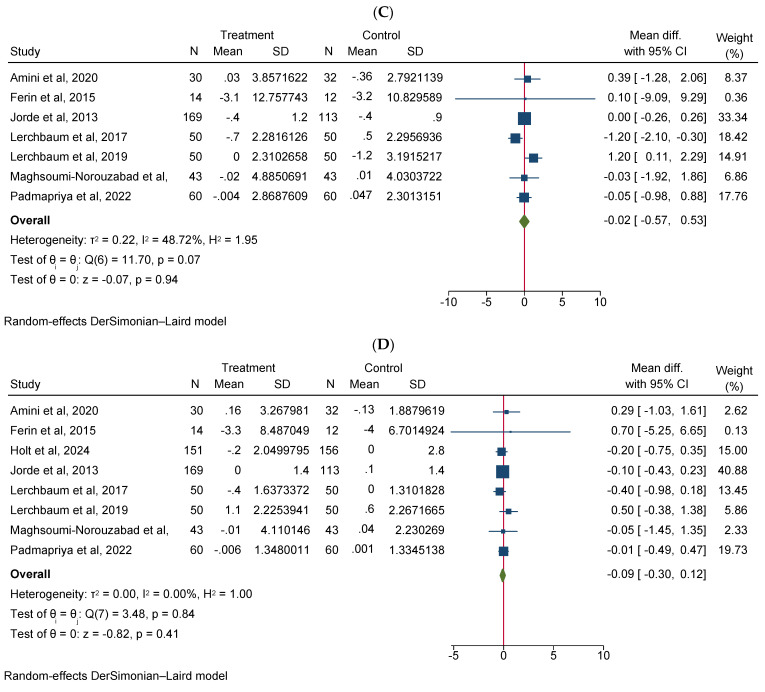

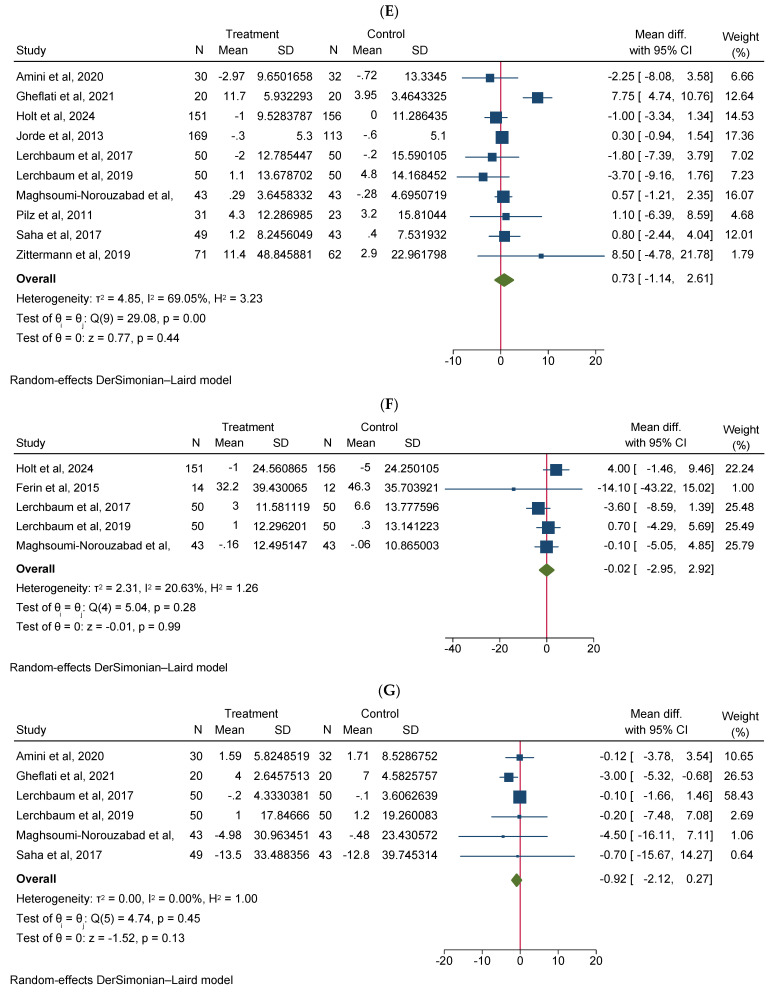
Meta-analysis of the effect of vitamin D supplementation on the endpoints: (**A**) total testosterone, (**B**) free testosterone, (**C**) FSH, (**D**) LH, (**E**) SHBG, (**F**) estradiol, and (**G**) FAI.

Vitamin D supplementation significantly increased total testosterone levels (WMD 0.38, 95% CI 0.06–0.70, n = 15, I^2^ = 67.03) (Figure 2A). Our subgroup analysis indicated that this effect was significant for durations of more than 12 weeks and vitamin D supplementation of more than 4000 IU/day compared to durations of 12 weeks or less and the supplementation of 4000 IU/day or less, respectively. We also observed in our subgroups that senior adults showed a significant increase in total testosterone after vitamin D supplementation compared to other age groups (Table 2). Our meta-analysis results indicated that vitamin D supplementation did not show any significant impact on other reproductive hormone parameters, such as free testosterone (WMD 0.00, 95% CI −0.02–0.03, n = 9, I^2^ = 48.12) (Figure 2B), FSH (WMD −0.02, 95% CI −0.57–0.53, n = 7, I^2^ = 48.72) (Figure 2C), LH (WMD −0.09, 95% CI −0.30–0.12, n = 8, I^2^ = 0.00) (Figure 2D), SHBG (WMD 0.73, 95% CI −1.14–2.61, n = 10, I^2^ = 69.05) (Figure 2E), estradiol (WMD −0.02, 95% CI −2.95–2.92, n = 5, I^2^ = 20.63) (Figure 2F), and FAI (WMD −0.92, 95% CI −2.12–0.27, n = 6, I^2^ = 0.00) (Figure 2G). However, our dose–response analysis indicated that there was an opposite correlation between the vitamin D dose and FSH levels, and there was also an opposite correlation between the duration of vitamin D supplementation and FAI levels. Additionally, we observed in our dose–response meta-analysis that a rise in the duration of vitamin D supplementation significantly correlated with free testosterone levels (Supplementary File S2).

Our subgroup analysis of these variables did not show any significant changes in the results, except in FAI. Interestingly, we observed that in infertile men or men with reproductive disorders, vitamin D supplementation significantly decreased FAI scores compared to healthy males. Moreover, FAI scores also significantly decreased in vitamin D-deficient participants, when vitamin D supplementation exceeded 4000 IU/day compared to non-deficient males, and in those patients receiving less than or equal to 4000 IU/day of vitamin D supplementation, respectively (Table 2).

A visual examination of Begg’s funnel plots indicated no potential publication biases for any of the variables of interest. These findings were further validated by Begg’s rank correlation and Egger’s regression asymmetry tests. We also performed sensitivity analyses for each individual variable. The leave-one-out sensitivity analyses did not show significant changes after the exclusion of each study for all variables (Supplementary File S2).

### 3.3. Summary of Quality Judgment and Certainty of Evidence

**Figure 3:**
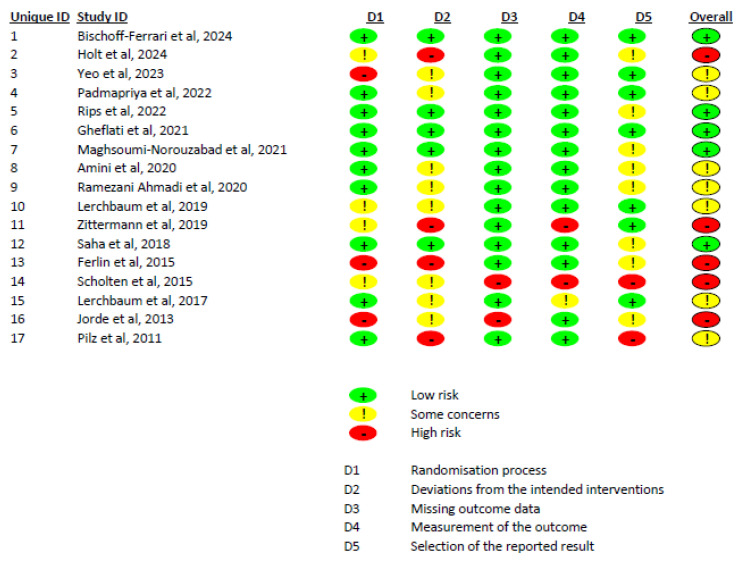
A summary of the risk of bias of the included studies.

The Cochrane risk of bias assessment, based on the ROB 2.0 tool, indicated that out of 17 included studies, 5 had a low risk of bias, 7 had some concerns, and 5 had a high risk of bias (Figure 3). We also evaluated the certainty of evidence for variables based on the GRADE tool. Total testosterone, free testosterone, free FAI, FSH, LH, and estradiol had moderate certainty of evidence, while SHBG had low certainty of evidence (Supplementary File S3).

## 4. Discussion

The correlation between vitamin D supplementation and androgens and related reproductive hormones is still debated. This meta-analysis is the first comprehensive review to gauge the effect of vitamin D on these hormones in men. Although previous reviews exist [43,44], they lack the breadth of evidence covered here. Our analysis, which included data from 17 clinical trials, found that vitamin D supplementation significantly increased total testosterone levels. However, it did not affect other parameters, such as free testosterone, FSH, LH, SHBG, FAI, and estradiol.

Our research offers contemporary understandings of the relationship between vitamin D supplementation and total testosterone levels by incorporating data from 17 trials, a significant increase compared to the 8 trials included in the prior meta-analysis by Hosseini Marnani et al. [43]. This expanded dataset allowed us to observe a notable correlation between vitamin D supplementation and increased total testosterone levels. These findings contrast with earlier systematic reviews and meta-analyses, which did not find a significant association, highlighting the importance of including a larger number of trials to obtain more comprehensive results. However, our meta-analysis findings also endorse earlier research, including previous systematic reviews and meta-analyses, that have identified a positive link between serum 25(OH) vitamin D levels and total testosterone [45]. This consistency in findings reinforces the significant positive correlation between these two biomarkers, which is observed in observational studies. Observational studies also advocate that there is a greater prevalence or jeopardy of testosterone deficiency in men with vitamin D deficiency [46,47,48]. Our findings are also supported by our subgroup analysis, which indicate that, compared to a lower dose and shorter duration of treatment, a higher dose and longer duration of vitamin D supplementation significantly increase total testosterone concentrations.

However, in our results, we failed to observe a significant impact of vitamin D supplementation on free testosterone. Following vitamin D supplementation, the observed increase in total testosterone levels but not in free testosterone levels can be attributed to the distinct nature and regulation of these two forms of testosterone [49]. Total testosterone encompasses both bound and unbound (free) testosterone [50]. Most testosterone in the blood is bound to SHBG or albumin, with only a small fraction circulating as free testosterone [51]. Vitamin D supplementation may influence total testosterone levels through mechanisms that increase the overall production of testosterone by the testes [46,52]. This increase, however, does not necessarily translate to a significant change in free testosterone levels. The absence of significant alteration in free testosterone concentrations could be due to several factors. For example, in (i) Binding Proteins, an increase in total testosterone might be accompanied by a rise in SHBG, which in turn binds to testosterone and regulates the quantity of free testosterone [53]. In our findings, we observed a trend towards an increase in SHBG; however, this trend was not statistically significant. Regarding (ii) feedback mechanisms, the endocrine system has complex feedback mechanisms with which to maintain homeostasis. Any increase in free testosterone might be quickly counteracted by the body to prevent potential androgen excesses, leading to a stable free testosterone concentration [54]. Regarding (iii) duration and dosage, the studies included varied in terms of duration and the dosage of vitamin D supplementation. While significant increases in total testosterone were observed with longer durations and higher doses of vitamin D, these conditions may not have been sufficient to alter free testosterone levels within the study periods.

The beneficial effects of vitamin D3 on increasing total testosterone can be attributed to its influence on testicular health [55]. Vitamin D3 supplementation has been shown to partially reverse testicular pathology by reducing fibrosis and apoptosis [56]. This is achieved through the downregulation of nuclear factor kappa B (NF-κB) and transforming growth factor beta 1 (TGF-β1) with testicular damage [57,58]. Additionally, vitamin D3 enhances the expression of peroxisome proliferator-activated receptor gamma (PPAR-γ), which may block the expression of both abovementioned mediators, thereby improving testicular function [52]. The interaction between PPAR-γ and vitamin D receptors with the retinoid X receptor also contributes to these protective effects [59]. Furthermore, experimental studies have demonstrated that vitamin D3 reduces TGF-β1 levels in the testes, reinforcing its role in mitigating fibrosis and supporting overall testicular health [60]. In addition, vitamin D supplementation might positively influence total testosterone levels due to its significant antioxidant properties [61]. Research indicates that oxidative stress can lead to decreased testosterone levels, suggesting that managing and reducing oxidative stress is crucial for maintaining healthy testosterone levels [62]. While vitamin D supplementation is less likely to cause toxic increases in endogenous testosterone, caution is still advised due to the potential adverse effects of elevated testosterone levels on various organs [63,64].

Our meta-analysis indicated that vitamin D supplementation did not significantly affect FSH, LH, SHBG, estradiol, or FAI scores. However, the dose–response analysis revealed nuanced relationships. An opposite link between vitamin D dose and FSH levels, as well as between supplementation duration and FAI levels, was observed. Notably, an increase in supplementation duration was significantly correlated with higher free testosterone levels. Subgroup analysis provided further interesting observations. Vitamin D supplementation significantly decreased FAI scores in specific subgroups: in infertile men or men with reproductive disorders, FAI significantly decreased compared to healthy males. Moreover, in vitamin D-deficient participants, FAI scores significantly decreased compared to non-deficient males. Additionally, in men who take a high dosage of vitamin D (>4000 IU/day), the FAI scores significantly decreased compared to those receiving ≤4000 IU/day of vitamin D supplementation. These subgroup results highlight the potential importance of the baseline vitamin D level and the dosage of supplementation regarding the outcomes of hormone parameters. These findings underscore the complexity of vitamin D’s contribution to endocrine function and suggest, that while overall effects on reproductive hormones might be minimal, specific subgroups, particularly those with existing deficiencies or reproductive issues, might benefit more significantly from supplementation rates. Further research should aim to elucidate the mechanisms behind these subgroup differences and explore optimal dosing strategies for different populations.

This meta-analysis has notable strengths and some limitations. A key strength is the use of RCTs to examine the connection between vitamin D supplementation and androgens and reproductive hormones. We thoroughly examined sources of heterogeneity among the included studies using meta-regression and subgroup analyses based on intervention duration, participant age, and vitamin D dosage. We also conducted analyses for publication bias, dose–response relationships, and sensitivity. Additionally, the generalizability of our results is bolstered by the inclusion of studies from various regions globally. The GRADE method was employed to evaluate the certainty of evidence, with most results showing moderate certainty. However, there were some limitations, such as the qualitative nature of our quality assessments and the lack of evaluation for inter-rater reliability. The small number of studies available for certain subgroup analyses and the high heterogeneity and risk of bias in the included studies also posed challenges.

## 5. Conclusions

Our meta-analysis of 17 RCTs involving 1774 men suggests that vitamin D supplementation may increase total testosterone levels particularly, with doses exceeding 4000 IU/day and durations longer than 12 weeks. The effect appears more pronounced in older adults. However, no significant impact was observed on other reproductive hormones, including free testosterone, FSH, LH, SHBG, estradiol, and FAI scores, except for an opposite correlation between vitamin D dose and FSH levels, and between supplementation duration and FAI scores. In infertile men or those with reproductive disorders, vitamin D significantly decreased FAI compared to healthy males. While these findings signify a potential use for vitamin D supplementation, supporting testosterone levels in men, caution is warranted given the relatively small sample sizes of the meta-analyzed RCTs and the wide age range of participants (20–74 years), which may limit the generalizability of our results. Larger, well-powered RCTs are necessary to confirm the impacts of vitamin D on testosterone and other reproductive hormones in diverse male populations, especially in post-intervention evaluation.

## Figures and Tables

**Table 1:** Main characteristics of included studies.

Study (Ref)	Country	Sample Size		Subjects	Vitamin D Dosage (IU/Day)	Duration (Week)	Age (Years)		BMI (kg/m^2^)		Main Outcome
		Intervention	Control				Intervention Mean ± SD	Placebo Mean ± SD	Intervention Mean ± SD	Placebo Mean ± SD	
Bischoff-Ferrari et al., 2024 [28]	Switzerland	**23**	**23**	Hypogonadal men	800	48	72.0 ± 5.9	72.4 ± 5.9	27.5 ± 2.9	26.1 ± 3.0	↔TT
Holt et al., 2024 [31]	Denmark	151	156	Infertile men	1400	21	35.0 ± 6.0	35.0 ± 7.0	26.3 ± 4.0	26.4 ± 4.8	↔TT, ↔LH, ↔TT/LH, ↔SHBG, ↔FT, ↔E, ↔FE, ↔T/E
Yeo et al., 2023 [41]	Korea	29	28	Vitamin D-deficient men	1600	36	65.07 ± 8.46	63.19 ± 6.19	25.48 ± 2.22	25.01 ± 3.27	↔TT
Padmapriya et al., 2022 [35]	India	60	60	Infertile men	4000	10	-	-	-	-	↔FSH, ↔LH
Rips et al., 2022 [38]	Estonia	27	26	Physically active male	1200	28	20.8 ± 1.7	21.2 ± 2.0	22.7 ± 2.4	23.2 ± 2.6	↔TT
Gheflati et al., 2021 [30]	Iran	20	20	Infertile men	7140	12	32.30 ± 1.30	33.00 ± 1.22	24.47 ± 0.86	26.34 ± 0.97	↔TT, ↔SHBG, ↔FAI
Maghsoumi-Norouzabad et al., 2021 [24]	Iran	43	43	Infertile men	4000	12	35.13 ± 5.51	34.44 ± 5.07	28.40 ± 2.96	27.95 ± 2.51	↔FSH, ↔LH, ↔TT, ↔SHBG, ↔E, ↔FAI, ↔T/E, ↑T/LH
Amini et al., 2020 [27]	Iran	30	32	Infertile men	7140	12	34.37 ± 4.83	34.86 ± 4.65	25.69 ± 1.94	25.47 ± 1.90	SHBG, ↔FAI, ↔TT, ↔FSH, ↔LH, ↔FT
Ramezani Ahmadi et al., 2020 [37]	Iran	20	20	Active healthy males	2000	12	23.7 ± 2.55	24.75 ± 4.15	23.77 ± 3.90	22.46 ± 3.03	TT
Lerchbaum et al., 2019 [34]	Austria	47	47	Healthy Males	2850	12	48.0 ± 14.0	50 ± 12.59	28.4 ± 4.22	29.4 ± 6.37	↔TT, ↔FT, ↔FSH, ↔LH, ↔SHBG, ↔E, ↔FAI
Zittermann et al., 2019 [42]	Germany	71	62	Patients with advanced heart failure (HF)	4000	48	55 ± 9.9	51.1 ± 10.5	29.2 ± 4.6	29.5 ± 5.3	↔TT, ↔FT, ↔SHBG
Saha et al., 2018 [39]	India	49	43	Vitamin D-deficient young males	8500	24	20.2 ± 2.2	20.2 ± 2.1	23.1 ± 3.4	23.4 ± 3.4	↔TT, ↔SHBG, ↔FAI
Lerchbaum et al., 2017 [33]	Austria	49	49	Healthy males	2850	12	34 ± 16.29	38 ± 17.77	25 ± 3.18	25.2 ± 3.48	↔TT, ↔FT, ↔FSH, ↔LH, ↔FAI, ↔SHBG
Ferlin et al., 2015 [29]	Italy	127	60	Non-mosaic KS patients	580	96	31.5 ± 8.5	30.9 ± 8.4	26.0 ± 4.6	23.6 ± 3.7	↔TT, ↔FSH, ↔LH, ↔E
Scholten et al., 2015 [40]	United States	11	12	Male adult athletes	4000	8	32.8 ± 5.63	29.9 ± 5.19	23.4 ± 5.96	26.2 ± 15.58	↔TT
Jorde et al., 2013 [32]	Norway	169	113	Healthy males	5700	48	50.0 ± 10.6	52.0 ± 10.9	31.1 ± 4.8	30.2 ± 4.6	↔TT, ↔FT, ↔FSH, ↔LH, ↔SHBG
Pilz et al., 2011 [36]	Germany	31	23	Overweight men	3332	48	49.4 ± 10.2	46.8 ± 12.0	33.1 ± 3.9	32.5 ± 3.8	↑TT, ↑FT

↑ this symbol is a sign of increasing variables in the intervention group. ↔ this sign indicates that there is no difference between the two groups. NR: not reported. E: estradiol. FE: free estradiol. T: testosterone. SHBG: sex-hormone-binding globulin. DHEA: dehydroepiandrosterone. FSH: follicle-stimulating hormone. LH: luteinizing hormone. OAT: oligoasthenoteratozoospermia. TT: total testosterone. FT: free testosterone. FAI: free androgen index.

**Table 2:** Subgroup analysis of interested variables.

Variable	Subgrouped by		No. of Studies	Effect Size WMD	95% CI	I^2^ (%)	*p* for Heterogeneity
**Estradiol**	Duration	≤12 weeks	3	−1.00	−3.87, 1.88	0.00	0.45
		>12 weeks	2	0.83	−12.64, 14.31	30.25	0.23
FAI	Health status	Infertile or reproductive disorder	4	**−2.11**	**−3.97**, −0.24 *	0.00	0.55
		Healthy	2	−0.11	−1.66, 1.45	0.00	0.94
	Vitamin D supplementation dosage	≤4000 IU/day	3	−0.18	−1.69, 1.34	0.00	0.76
		>4000 IU/day	3	**−2.15**	**−4.09**, −0.21 *	0.00	0.42
	Baseline vitamin D status	No deficiency	2	−0.10	−1.63, 1.42	0.00	0.98
		Deficient	4	**−2.21**	**−4.13**, −0.30 *	0.00	0.60
Free Testosterone	Health status	Infertile or reproductive disorder	6	−0.00	−0.06, 0.05	0.00	0.02
		Healthy	3	0.00	−0.02, 0.02	0.00	0.05
	Duration	≤12 weeks	4	−0.01	−0.03, 0.02	0.00	0.09
		>12 weeks	5	0.03	−0.03, 0.08	0.00	0.15
	Vitamin D supplementation dosage	≤4000 IU/day	6	−0.00	−0.01, 0.01	0.00	0.69
		>4000 IU/day	3	0.22	−0.50, 0.94	80.83	0.01
	Participant’s age	Young adults (20–30)	1	−0.03	−0.15, 0.09	-	-
		Middle-age adults (31–50)	6	0.00	−0.02, 0.03	0.00	0.08
		Senior adults (>50)	2	0.22	−0.30, 0.74	79.98	0.03
	Baseline vitamin D status	No deficiency	4	−0.00	−0.01, 0.01	0.00	0.72
		Deficient	4	−0.00	−0.09, 0.08	0.00	0.01
FSH	Health status	Infertile or reproductive disorder	5	0.41	−0.21, 1.02	0.00	0.53
		Healthy	2	−0.52	−1.69, 0.65	84.23	0.01
	Duration	≤12 weeks	5	0.01	−0.90, 0.93	65.41	0.02
		>12 weeks	2	−0.02	−0.26, 0.26	0.00	0.98
	Vitamin D supplementation dosage	≤4000 IU/day	6	0.01	−0.86, 0.88	56.77	0.04
		>4000 IU/day	1	0.00	−0.26, 0.26	-	-
	Baseline vitamin D status	No deficiency	5	−0.05	−0.75, 0.64	65.10	0.02
		Deficient	2	0.21	−1.05, 1.46	0.00	0.74
LH	Health status	Infertile or reproductive disorder	6	0.01	−0.30, 0.33	0.00	0.85
		Healthy	2	−0.17	−0.46, 0.11	0.00	0.38
	Duration	≤12 weeks	5	−0.04	−0.37, 0.28	0.00	0.53
		>12 weeks	3	−0.12	−0.41, 0.16	0.00	0.92
	Vitamin D supplementation dosage	≤4000 IU/day	6	−0.10	−0.38, 0.18	0.00	0.68
		>4000 IU/day	2	−0.08	−0.40, 0.25	0.00	0.57
	Baseline vitamin D status	No deficiency	6	−0.10	−0.32, 0.12	0.00	0.68
		Deficient	2	0.13	−0.83, 1.09	0.00	0.73
SHBG	Health status	Infertile or reproductive disorder	6	0.72	−2.52, 3.96	81.05	0.00
		Healthy	4	0.33	−0.80, 1.47	0.00	0.55
	Duration	≤12 weeks	6	0.70	−2.55, 3.95	79.32	0.00
		>12 weeks	4	0.09	−0.99, 1.18	0.00	0.47
	Vitamin D supplementation dosage	≤4000 IU/day	6	−0.20	−1.50, 1.11	0.00	0.44
		>4000 IU/day	4	1.91	−1.98, 5.80	86.11	0.00
	Participant’s age	Young adults (20–30)	1	0.80	−2.44, 4.04	-	-
		Middle-age Adults (31–50)	8	0.54	−1.59, 2.66	74.74	0.00
		Senior adults (>50)	1	8.50	−4.78, 21.78	-	-
	Baseline vitamin D status	No deficiency	4	−0.19	−1.25, 0.87	0.00	0.40
		Deficient	6	2.26	−1.09, 5.60	75.04	0.00
Total Testosterone	Health status	Infertile or reproductive disorder	8	0.13	−0.06, 0.33	34.66	0.15
		Healthy	7	1.00	−0.11, 2.12	74.50	0.00
	Duration	≤12 weeks	7	0.68	−0.13, 1.48	83.69	0.00
		>12 weeks	**8**	**0.13**	**0.04**, 0.22 *	0.00	0.62
	Vitamin D supplementation dosage	≤4000 IU/day	9	0.75	−0.34, 1.85	75.40	0.00
		>4000 IU/day	**6**	**0.12**	**0.04**, 0.20 *	0.00	0.83
	Participant’s age	Young adults (20–30)	2	0.62	−1.14, 2.39	0.00	0.75
		Middle-age adults (31–50)	11	0.54	−0.01, 1.10	75.78	0.00
		Senior adults (>50)	**2**	**0.12**	**0.03**, 0.21 *	0.00	0.99
	Baseline vitamin D status	No deficiency	7	0.57	−0.71, 1.85	80.47	0.00
		Deficient	8	0.16	−0.00, 0.33	23.69	0.24

Bold indicates statistical significance (*p* < 0.05). CI: confidence interval; WMD: weighted mean difference. * Statistically significant at *p* < 0.05.

## Data Availability

All data are available within the manuscript and its Supplementary Files.

## Supplementary Materials

The following supporting information can be downloaded at: https://www.mdpi.com/article/10.3390/diseases12100228/s1, Supplementary File S1: The detailed search strategy in all databases [65,66,67,68,69,70,71,72,73,74,75,76]; Supplementary File S2: Summary of [A] publication bias, [B] sensitivity analysis, [C] dose-response analysis, and [D] meta-regression analysis; Supplementary File S3: Summary of certainty of evidence according to the GRADE approach.
