# Supplementary material for: The Impact of Vitamin D on Androgens and Anabolic Steroids among Adult Males: A Meta-Analytic Review
Source: Diseases. 2024 Sep 25;12(10):228. doi: 10.3390/diseases12100228 (PMC11506788; doi:10.3390/diseases12100228)
Supplement: Supplementary file 1 [file diseases-12-00228-s001.zip › Supplementary File S1.pdf]

Supplementary File S1. The detailed search strategy in all databases.

Exploring the Impact of Vitamin D on Androgens and Anabolic Steroids Among Adult Males: A Systematic Review and Dose-Response Meta-Analytic Review of Randomized Controlled Trials

Groups	Descriptors
Outcome	“Androgens” OR “Anabolic Androgenic Steroids” OR “Male Sex Hormone” OR “Testosterone” OR “Sex Hormone-Binding Globulin” OR “SHBG” OR “Dehydroepiandrosterone” OR “DHEA”
Exposure	Vitamin D OR Cholecalciferol OR Hydroxycholecalciferols OR Ergocalciferols OR 25-Hydroxyvitamin D 2 OR Dihydrotachysterol OR Calcitriol OR 1 alpha,25-Dihydroxyvitamin D3 OR 1,25 Dihydroxyvitamin D3 OR Vitamin D3 OR 1 alpha,25-Dihydroxycholecalciferol OR 1,25(OH)2-20epi-D3 OR 1,25(OH)-D3
Setting	Randomized controlled trial OR controlled clinical trial OR randomized controlled trials OR random allocation OR double blind method OR single blind method OR clinical trial OR clinical trials OR placebos OR placebo OR random

PUBMED

Number of localized studies: 1965
Limits: humans
Number of studies after applying limits: 1892

	Descriptors	Number of studies reached
**#1**	"androgen s"[All Fields] OR "androgene"[All Fields] OR "androgenes"[All Fields] OR "androgenic"[All Fields] OR "androgenicity"[All Fields] OR "androgenized"[All Fields] OR "androgenizing"[All Fields] OR "androgenous"[All Fields] OR "androgens"[Pharmacological Action] OR "androgens"[MeSH Terms] OR "androgens"[All Fields] OR "androgen"[All Fields] OR "virilism"[MeSH Terms] OR "virilism"[All Fields] OR "androgenization"[All Fields] OR ("testosterone congeners"[MeSH Terms] OR ("testosterone"[All Fields] AND "congeners"[All Fields]) OR "testosterone congeners"[All Fields] OR ("anabolic"[All Fields] AND "androgenic"[All Fields] AND "steroids"[All Fields]) OR "anabolic androgenic steroids"[All Fields] OR "anabolic androgenic steroids"[MeSH Terms]) OR (("male"[MeSH Terms] OR "male"[All Fields] OR ("male"[All Fields] AND "sex"[All Fields]) OR "male sex"[All Fields]) AND ("hormon"[All Fields] OR "hormonal"[All Fields] OR "hormonally"[All Fields] OR "hormonals"[All Fields] OR "hormone s"[All Fields] OR "hormones"[Pharmacological Action] OR "hormones"[MeSH Terms] OR "hormones"[All Fields] OR "hormone"[All Fields] OR "hormons"[All Fields])) OR ("testosterone"[MeSH Terms] OR "testosterone"[All Fields] OR "testosteron"[All Fields] OR "testosterones"[All Fields] OR "testosterone s"[All Fields]) OR ("sex hormone binding globulin"[MeSH Terms] OR ("sex"[All Fields] AND "hormone binding"[All Fields] AND "globulin"[All Fields]) OR "sex hormone binding globulin"[All	814884

	Fields] OR ("sex"[All Fields] AND "hormone"[All Fields] AND "binding"[All Fields] AND "globulin"[All Fields]) OR "sex hormone binding globulin"[All Fields]) OR ("sex hormone binding globulin"[MeSH Terms] OR ("sex"[All Fields] AND "hormone binding"[All Fields] AND "globulin"[All Fields]) OR "sex hormone binding globulin"[All Fields] OR "shbg"[All Fields]) OR ("dehydroepiandrosteron"[All Fields] OR "dehydroepiandrosterone"[MeSH Terms] OR "dehydroepiandrosterone"[All Fields]) OR ("dehydroepiandrosterone"[MeSH Terms] OR "dehydroepiandrosterone"[All Fields] OR "dhea"[All Fields])	
**#2**	((((((((((("Vitamin D"[Mesh]) OR "Cholecalciferol"[Mesh]) OR "Hydroxycholecalciferols"[Mesh]) OR "Ergocalciferols"[Mesh]) OR "25-Hydroxyvitamin D 2"[Mesh]) OR "Dihydrotachysterol"[Mesh]) OR "Calcitriol"[Mesh]) OR 1 alpha,25-Dihydroxyvitamin D3) OR 1,25 Dihydroxyvitamin D3) OR Vitamin D3) OR 1 alpha,25-Dihydroxycholecalciferol)	82118
**#3**	((((((((("Randomized Controlled Trial"[Publication Type] OR "Controlled Clinical Trial"[Publication Type]) OR "Randomized Controlled Trials as Topic"[Mesh]) OR "Random Allocation"[Mesh]) OR "Double-Blind Method"[Mesh]) OR "Single-Blind Method"[Mesh]) OR "Clinical Trial"[Publication Type]) OR ("clinical trial"[Publication Type] OR "clinical trials as topic"[MeSH Terms] OR "clinical trials"[All Fields])) OR "Placebos"[Mesh]) OR ("placebos"[MeSH Terms] OR "placebos"[All Fields] OR "placebo"[All Fields])) OR ("random allocation"[MeSH Terms] OR ("random"[All Fields] AND "allocation"[All Fields]) OR "random allocation"[All Fields] OR "random"[All Fields])	2070071
**#4**	**#1** AND **#2** AND #3	1965

WEB OF SCIENCE

Number of localized studies: 442
Limits: documents types (articles)
Number of studies after applying limits: 286

	**Descriptors**	Number of studies reached
**#1**	TS=(Androgens) OR TS=(Anabolic Androgenic Steroids) OR TS=(Male Sex Hormone) OR TS=(Testosterone) OR TS=(Sex Hormone-Binding Globulin) OR TS=(SHBG) OR TS=(Dehydroepiandrosterone) OR TS=(DHEA)	234884
**#2**	TS=(“Vitamin D”) OR TS=(“Cholecalciferol”) OR TS=(“Hydroxycholecalciferols”) OR TS=(“Ergocalciferols”) OR TS=(“25-Hydroxyvitamin D 2”) OR TS=(“Dihydrotachysterol”) OR TS=(“Calcitriol”) OR TS=(“1 alpha,25-Dihydroxyvitamin D3”) OR TS=(“1,25 Dihydroxyvitamin D3”) OR TS=(“Vitamin D3”) OR TS=(“1 alpha,25-Dihydroxycholecalciferol”) OR TS=(“1,25(OH)2-20epi-D3”) OR TS=(“1,25(OH)-D3”)	131348
**#3**	TS=(Randomized controlled trial) OR TS=(controlled clinical trial) OR TS=(randomized controlled trials) OR TS=(random allocation) OR	2436205

	TS=(double blind method) OR TS=(single blind method) OR TS=(clinical trial) OR TS=(clinical trials) OR TS=(placebos) OR TS=(placebo) OR TS=(random)	
**#4**	**#1** AND **#2** AND **#3**	442

SCOPUS

Number of localized studies: 1904
Limits: *document type* (article and article in press)
Number of studies after applying limits: 961

	**Descriptors**	Number of studies reached
**#1**	(TITLE-ABS-KEY (androgens)) OR (TITLE-ABS-KEY (anabolic AND androgenic AND steroids)) OR (TITLE-ABS-KEY (male AND sex AND hormone)) OR (TITLE-ABS-KEY (testosterone)) OR (TITLE-ABS-KEY (sex AND hormone-binding AND globulin)) OR (TITLE-ABS-KEY (shbg)) OR (TITLE-ABS-KEY (dehydroepiandrosterone)) OR (TITLE-ABS-KEY (dhea))	317365
**#2**	((TITLE-ABS-KEY (vitamin AND d) OR TITLE-ABS-KEY (cholecalciferol) OR TITLE-ABS-KEY (hydroxycholecalciferols) OR TITLE-ABS-KEY (ergocalciferols) OR TITLE-ABS-KEY (25-hydroxyvitamin AND d 2))) OR ((TITLE-ABS-KEY (dihydrotachysterol) OR TITLE-ABS-KEY (calcitriol) OR TITLE-ABS-KEY (1 alpha,25-dihydroxyvitamin AND d3) OR TITLE-ABS-KEY (1,25 AND dihydroxyvitamin AND d3) OR TITLE-ABS-KEY (vitamin AND d3))) OR (TITLE-ABS-KEY (1 alpha,25-dihydroxycholecalciferol))	194451
**#3**	(TITLE-ABS-KEY (randomized AND controlled AND trial) OR TITLE-ABS-KEY (controlled AND clinical AND trial) OR TITLE-ABS-KEY (randomized AND controlled AND trials) OR TITLE-ABS-KEY (random AND allocation) OR TITLE-ABS-KEY (double AND blind AND method) OR TITLE-ABS-KEY (single AND blind AND method) OR TITLE-ABS-KEY (clinical AND trial) OR TITLE-ABS-KEY (clinical AND trials) OR TITLE-ABS-KEY (placebos) OR TITLE-ABS-KEY (placebo) OR TITLE-ABS-KEY (random))	4025719
**#4**	**#1** AND **#2** AND **#3**	1904

COCHRANE CENTRAL

Number of localized studies: 109
Limits: -
Number of studies after applying limits: 109

	**Descriptors**	Number of studies reached
**#1**	Me ("Androgens") or ("Anabolic Androgenic Steroids"):ti,ab,kw or ("Male Sex Hormone"):ti,ab,kw or ("Testosterone"):ti,ab,kw or ("Sex Hormone-Binding Globulin"):ti,ab,kw or ("SHBG"):ti,ab,kw or ("Dehydroepiandrosterone"):ti,ab,kw or ("DHEA"):ti,ab,kw	10853
**#2**	Me ("Vitamin D") or ("Cholecalciferol"):ti,ab,kw or ("Hydroxycholecalciferols"):ti,ab,kw or ("Ergocalciferols"):ti,ab,kw or ("25-Hydroxyvitamin D 2"):ti,ab,kw or ("Dihydrotachysterol"):ti,ab,kw or ("Calcitriol"):ti,ab,kw or ("1 alpha,25-Dihydroxyvitamin D3"):ti,ab,kw or ("1,25 Dihydroxyvitamin D3"):ti,ab,kw or ("Vitamin D3"):ti,ab,kw or ("1 alpha,25-Dihydroxycholecalciferol"):ti,ab,kw or ("1,25(OH)2-20epi-D3"):ti,ab,kw or ("1,25(OH)-D3"):ti,ab,kw	9159
**#3**	**#1** AND **#2**	109

EXCLUDED ARTICLES

	**Study**	**Reason**
1	Jensen et al, 2018 [1]	In combination with other interventions
2	Canguven et al, 2017 [2]	Not RCT
3	Chehsmazar et al, 2020 [3]	Conduct in both genders
4	Chen et al, 2019 [4]	Not RCT
5	Hejiboer et al, 2015[5]	Did not provide enough information
6	Kenny et al, 2003 [6]	Did not provide enough information
7	Maghsoumi-Norouzabad et al, 2022 [7]	Not relevant variables
8	Midttun et al, 2024 [8]	In combination with other interventions
9	Peppone et al, 2024 [9]	Not relevant variables
10	Tirabassi et al, 2017 [10]	Not RCT
11	Ulrich et al, 2021 [11]	Did not provide enough information
12	Zhang et al, 2023 [12]	Did not provide enough information

REFERENCES

1. Blomberg Jensen, M.; Lawaetz, J.G.; Petersen, J.H.; Juul, A.; Jørgensen, N. Effects of vitamin D supplementation on semen quality, reproductive hormones, and live birth rate: a randomized clinical trial. *The Journal of Clinical Endocrinology & Metabolism* **2018**, *103*, 870-881.
2. Canguven, O.; Talib, R.A.; El Ansari, W.; Yassin, D.-J.; Al Naimi, A. Vitamin D treatment improves levels of sexual hormones, metabolic parameters and erectile function in middle-aged vitamin D deficient men. *The Aging Male* **2017**, *20*, 9-16.
3. Chehsmazar, E.; Zarrati, M.; Yazdani, B.; Razmpoosh, E.; Hosseini, A.F.; Shidfar, F. The effect of vitamin D supplementation on serum concentrations of dehydroepiandrosterone, paraoxonase 1, apolipoproteins, free fatty acid and insulin in vitamin D deficient obese and overweight individuals under a low-calorie diet program: a randomized controlled trial. *Nutrition & Food Science* **2021**, *51*, 765-780.
4. Chen, C.; Zhai, H.; Cheng, J.; Weng, P.; Chen, Y.; Li, Q.; Wang, C.; Xia, F.; Wang, N.; Lu, Y. Causal link between vitamin D and total testosterone in men: a Mendelian randomization analysis. *The Journal of Clinical Endocrinology & Metabolism* **2019**, *104*, 3148-3156.
5. Heijboer, A.C.; Oosterwerff, M.; Schroten, N.F.; Eekhoff, E.M.; Chel, V.G.; de Boer, R.A.; Blankenstein, M.A.; Lips, P. Vitamin D supplementation and testosterone concentrations in male human subjects. *Clinical endocrinology* **2015**, *83*, 105-110.
6. Kenny, A.M.; Biskup, B.; Robbins, B.; Marcella, G.; Burleson, J.A. Effects of vitamin D supplementation on strength, physical function, and health perception in older, community-dwelling men. *Journal of the American Geriatrics Society* **2003**, *51*, 1762-1767.
7. Maghsoumi-Norouzabad, L.; Zare Javid, A.; Mansoori, A.; Dadfar, M.; Serajian, A. Vitamin D3 supplementation effects on spermatogram and oxidative stress biomarkers in asthenozoospermia infertile men: a randomized, triple-blind, placebo-controlled clinical trial. *Reproductive Sciences* **2022**, 1-13.
8. Midttun, M.; Overgaard, K.; Zerahn, B.; Pedersen, M.; Rashid, A.; Østergren, P.B.; Paulin, T.K.; Pødenphanth, T.W.; Karlsson, L.K.; Rosendahl, E. Beneficial effects of exercise, testosterone, vitamin D, calcium and protein in older men—A randomized clinical trial. *Journal of Cachexia, Sarcopenia and Muscle* **2024**.
9. Peppone, L.J.; Kleckner, A.S.; Fung, C.; Puzas, J.E.; Reschke, J.E.; Culakova, E.; Inglis, J.; Kamen, C.; Friedberg, J.W.; Janelsins, M. High-dose vitamin D to attenuate bone loss in patients with prostate cancer on androgen deprivation therapy: A phase 2 RCT. *Cancer* **2024**.
10. Tirabassi, G.; Cutini, M.; Salvio, G.; Cerqueni, G.; Lenzi, A.; Balercia, G. Influence of vitamin D levels on the cardiovascular profile of hypogonadal men. *Journal of endocrinological investigation* **2017**, *40*, 1007-1014.
11. Ulrich, C.; Trojanowicz, B.; Fiedler, R.; Kraus, F.B.; Stangl, G.I.; Girndt, M.; Seibert, E. Serum testosterone levels are not modified by vitamin d supplementation in dialysis patients and healthy subjects. *Nephron* **2021**, *145*, 481-485.
12. Zhang, X.-L.; Zhang, Q.; Zhang, X.; Gu, Q.-W.; Pan, J.-J.; Pei, Y.-F.; Li, J.-F.; Jiang, F.; Diao, L.-J.; Zhou, H.-M. Effect of vitamin D3 supplementation in winter on physical performance of university students: a one-month randomized controlled trial. *Journal of the International Society of Sports Nutrition* **2023**, *20*, 2258850.
