# Supplementary material for: The Impact of Vitamin D on Androgens and Anabolic Steroids among Adult Males: A Meta-Analytic Review
Source: Diseases. 2024 Sep 25;12(10):228. doi: 10.3390/diseases12100228 (PMC11506788; doi:10.3390/diseases12100228)
Supplement: Supplementary file 1 [file diseases-12-00228-s001.zip › Supplementary File S2.pdf]

Supplementary File S2. Summary of **[A]** publication bias, **[B]** sensitivity analysis, **[C]** dose-response analysis, and **[D]** meta-regression analysis.

[A] Publication Bias

Variables	Publication Bias (p values)	
	Egger's test	Begg's test
Total Testosterone	0.347	0.553
Estradiol	1.000	0.556
FAI	0.452	0.629
Free Testosterone	0.348	0.480
FSH	0.764	0.893
LH	0.711	0.194
SHBG	0.592	0.776

[B] Leave-One-Out Sensitivity Analysis

Variables	Study	Effect size after remove of the study
Total Testosterone	-	-
Estradiol	-	-
FAI	-	-
Free Testosterone	-	-
FSH	-	-
LH	-	-
SHBG	-	-

[C] Dose-Response Analysis

Variables	Dose response			
	Duration		Dose	
	Coefficient	P value	Coefficient	P value
Total Testosterone	0.752	0.708	0.016	0.858
Estradiol	-11.922	0.054	-0.742	0.892
FAI	**-904.628**	**0.019***	-19.089	0.132
Free Testosterone	**0.499**	**0.036***	-0.044	0.242
FSH	0.419	0.464	**-2.058**	**0.001***
LH	-1.248	0.231	-0.019	0.066
SHBG	-20.751	0.393	-2.518	0.139

Bolded values indicate statistical significance.

[D] Meta-Regression Analysis

Variables	Meta-Regression			
	Duration		Dose	
	Coefficient	P value	Coefficient	P value
Total Testosterone	-8.273	0.152	-514.532	0.863
Estradiol	-4.244	0.082	98.0219	0.377
FAI	-0.003	0.995	-3472.647	0.491
Free Testosterone	4.365	0.872	**24281.330**	**0.009***
FSH	0.978	0.962	236.229	0.980
LH	47.152	0.113	-6624.799	0.659
SHBG	3.929	0.072	1826.225	0.221

Bolded values indicate statistical significance
