# Supplementary material for: The Impact of Vitamin D on Androgens and Anabolic Steroids among Adult Males: A Meta-Analytic Review
Source: Diseases. 2024 Sep 25;12(10):228. doi: 10.3390/diseases12100228 (PMC11506788; doi:10.3390/diseases12100228)
Supplement: Supplementary file 1 [file diseases-12-00228-s001.zip › Supplementary File S3.pdf]

Supplementary File S3. Summary of certainty of evidence according to the GRADE approach.

Certainty assessment							№ of patients		Effect		Certainty
№ of studies	Study design	Risk of bias	Inconsistency	Indirectness	Imprecision	Other considerations	Vitamin D supplementation	Placebo	Relative (95% CI)	Absolute (95% CI)	
**Total testosterone**											
15	randomised trials	not serious	serious[a]	not serious	not serious	none	877	737	-	MD **0.38 higher** (0.06 higher to 0.7 higher)	⊕⊕⊕◯ Moderate
**Free testosterone**											
9	randomised trials	not serious	not serious	not serious	serious[b]	none	591	525	-	MD **0** (0.02 lower to 0.03 higher)	⊕⊕⊕◯ Moderate
**FSH**											
7	randomised trials	not serious	not serious	not serious	serious[b]	none	416	360	-	MD **0.02 lower** (0.57 lower to 0.53 higher)	⊕⊕⊕◯ Moderate
**LH**											
8	randomised trials	not serious	not serious	not serious	serious[b]	none	567	516	-	MD **0.09 lower** (0.3 lower to 0.12 higher)	⊕⊕⊕◯ Moderate
**SHBG**											
10	randomised trials	not serious	serious[a]	not serious	serious[b]	none	664	592	-	MD **0.73 higher** (1.14 lower to 2.61 higher)	⊕⊕◯◯ Low

Certainty assessment							№ of patients		Effect		Certainty
№ of studies	Study design	Risk of bias	Inconsistency	Indirectness	Imprecision	Other considerations	Vitamin D supplementation	Placebo	Relative (95% CI)	Absolute (95% CI)	
**Estradiol**											
5	randomised trials	not serious	not serious	not serious	serious[b]	none	308	311	-	MD **0.02 lower** (2.95 lower to 2.92 higher)	⨁⨁⨁◯ Moderate
**Free Androgen index (FAI)**											
6	randomised trials	not serious	not serious	not serious	serious[b]	none	242	238	-	**0.92 lower** (2.12 lower to 0.27 higher)	⨁⨁⨁◯ Moderate

CI: confidence interval; **MD:** mean difference

Explanations

a. Serious inconsistency since I^2>60%. Downgraded.

b. Serious Imprecision since the meta-analysis result was not statistically significant. Downgraded
